# Toxicity treatment of tobacco wastes using experimental design by filamentous fungi

**DOI:** 10.1016/j.heliyon.2021.e06144

**Published:** 2021-02-08

**Authors:** William Bartolomeu Medeiros, Jaqueline Bail, Michel Rodrigo Zambrano Passarini, Rafaella Costa Bonugli-Santos

**Affiliations:** Latin American Institute of Science's Life and Nature – ILACVN, UNILA – Federal University of Latin American Integration, Foz do Iguassu, Paraná, Brazil

**Keywords:** Cigarette, Toxicity, Bioremediation, Fractional factorial experimental design, Central composite design

## Abstract

Cigarette product waste contains toxic chemicals, including human carcinogens, which leach into and accumulate in the environment and represent a current environmental problem neglected for too long. This study aimed to select filamentous fungi capable of decreasing tobacco extract toxicity as an alternative to a future bioremediation process. The 38 isolates obtained from Culture collection of microorganisms to biotechnological and environmental importance – CCMIBA (Brazil) were cultivated in yeast extract (10 g.L^−1^) and dextrose (10 g.L^−1^) containing cigarette tobacco extract (200 mL.L^−1^) for seven days at 28 °C on a rotary shaker at 150 rpm. The fungal growth rate was determined to infer fungal tolerance to tobacco extract, and supernatants from cultivated fungi were used to run the toxicity test using *Allium cepa* assay. The *Fusarium* sp. strain I.17, isolated from cigarette waste, was the only lineage capable of growing in 20% (v/v) of cigarette tobacco extract, allowed the onions to root, and was selected for optimization. Initially, for the experimental design to selected fungus, a fractional factorial experimental design 2^5−1^ was used to examine the effects of yeast extract, cigarette tobacco extract concentration, dextrose, copper sulfate and pH fungal cultivation. The supernatants of these assays were used to run the toxicity test, and yeast extract and copper sulfate were statistically significant in the fungal growth for the decreasing toxicity process and this variable as were select to central composite design. The highest concentration of yeast extract negatively influenced the toxicity decrease, 0.5% of yeast extract in the culture media is the maximum concentration to achieve the best result and to copper sulfate the best result was using 10 μmol.L^−1^. In conclusion, the experimental design optimized more than seven times the efficiency of tobacco toxicity reducing, resulting in more than 50% of onion root growth, demonstrating the methodology success. And ITS region was used to taxonomy and molecular phylogeny of the isolate *Fusarium* sp. strain I.17. These results suggest that *Fusarium* sp. strain I.17 can be used as a potential microorganism to toxicity treatment of cigarette wastes, minimizing the environmental impact of direct burning.

## 1 Introduction

The cigarette is the most common way to make use of tobacco. It consists of a small amount of dry and cut leaves of a *Nicotiana* genus plant, known as tobacco. Apart from the nicotine, cigarette smoke contains about 5,000 chemical compounds that are considered harmful, such as carbon monoxide, and polycyclic aromatic hydrocarbons—PAH (Moreira, 2007; Pedroso, 2003; Rosa, 2013). Since many studies shown the potential of cigarettes to affect human health, cigarette industries have been trying to minimize the toxicity of this product by applying a filter in cigarettes to adsorb particulate matter of the smoke. Also, improvements in the leaf's maturation have been made to decrease nicotine concentration (Rosa, 2013).

Cigarette butts and other post-consumer products from tobacco use are the most common waste elements picked up worldwide during environmental clean-ups (Curtis et al., 2017). It results in a problem for discards. These toxic components are left in the environment without any specific treatment, and clean-up and disposal are not available borne by manufacturers, distributors, or users of tobacco products. Direct burning or composting for use as organic fertilizer are the conventional methods for dealing with cigarette waste. However, some chemical compounds, such as nicotine, can remain (Liu et al., 2015). In addition, approximately 4 million packs of Paraguayan smuggled cigarettes are illegally brought into Brazil, which do not follow any quality process in production (Trilha, 2009; Rosa, 2013). The seizure of this product by the Federal Revenue Service of Brazil reduces the impact on the Brazilian population health, along with reducing the negative impacts on the tobacco industry and, therefore, reducing economic damages in the country (Trilha, 2009). Despite that, once the smuggled cigarettes are seized, it needs to be destroyed, and this procedure generates high costs with transportation, storage, and correct final destination. Currently, the practice for the destruction of seized cigarettes has been incineration, a costly and non-ecological alternative (IDESF, 2015). In this sense, eco-friendly options are necessary to correct the destination of the seized cigarettes.

Microorganisms have been applied to clean up the polluted environment, either *in situ* or *ex-situ*. Bioremediation using microorganisms is a successful methodology to treat an environment contaminated by pesticides, effluents from textiles, heavy metals and derivatives of oil refining (Rhodes, 2015). Pedroso (2003) proposed using tobacco industrial waste as a substrate for the production of edible mushrooms, highlighting the potential of those organisms to resist the nicotine toxicity. Wang et al. (2009) showed, for example, the potential to treat an environment contaminated by nicotine applying *Agrobacterium* sp. strains. Nevertheless, efficient and applicable treatment is scarce in the literature. In this sense, the toxicological impact must be considered as one of the priorities. Almeida (2015) showed an alternative for treating cigarette waste using a composting process with sludge from industrial sewage treatment plants. However, according to the author, further studies are needed to ensure that the by-product generated after the composting process is not harmful to the environment and human health. Zheng et al. (2017) used *Rhizopus oryzae*, a filamentous fungus, to develop a process for pectinase production using tobacco wastewater as the sole substrate. However, as far as we know, there is no record in the literature for the toxicity treatment of cigarette waste using filamentous fungi. The innovation from our study concerning other records was the use of fungal cultivation evaluated directly in toxicity, which is the leading environmental problem. Our work uses as a highlight a statistical methodology that facilitates the study and allows, in the future, a more in-depth bioremediation study. Therefore, this study initially aimed to select filamentous fungi capable of decreasing cigarette toxicity and, secondly, to provide optimal cultivation conditions to enable use in a potential future application for correct and environmentally friendly disposal of seized cigarettes and other waste from tobacco.

## 2 Material and methods

### 2.1 Strains and culture conditions

The 38 fungal strains were obtained from the Culture collection of microorganisms to biotechnological and environmental importance – CCMIBA - UNILA, Parana state, Brazil (Supplementary material), since 19 fungi were previously isolated from Iguassu National Park and 19 isolated from cigarette samples, as described below. . The cigarettes used in this study were provided by Federal Revenue Service from Foz do Iguaçu city. To isolate fungi from cigarettes, about 10 g from massed cigarettes was agitated with 50 mL of saline solution (0.1 % NaCl) for 30 min, then 100 μl of this solution was inoculated by serial dilution in PDA media (potato 200 g.L^−1^, dextrose 20 g.L^−1^ and agar 20 g.L^−1^) and incubated at 28 °C. The isolates were purified and preserved in glycerol 20% at -80 °C.

The strains were reactivated on PDA media and incubated for seven days at 28 °C. Afterward, the inoculation process was followed as described by Pedroso (2003), with some modifications as follows: three discs with approximately 5 mm of diameter from each strain were used to inoculate 50 mL of YDF media (yeast extract 10 g.L^−1^, dextrose 10 g.L^−1^ and tobacco extract 200 mL.L^−1^). The cultivation was conducted at 28 °C on a rotary shaker at 150 rpm for seven days. According to Pedroso (2003), the tobacco extract was made by boiling cigarette waste (200 g) in 1 L of distilled water for 45 min and filtering it to separate solid matters. The liquid acquired was considered as tobacco extract to this study. After incubation, the mycelium was filtered and dried at 60 °C until reaching a constant weight, and the supernatant was used to carry on the toxicity assay.

### 2.2 Experimental design

The strategy used in the experimental design for *Fusarium* sp. (I.17) was composed of one fractional factorial experiment, one central composite design, and the validation assay.

Fractional Factorial design 2^5−1^ (Rodrigues and Iemma, 2005) was used to evaluate five independent factors (variables): tobacco extract, yeast extract, dextrose, copper sulfate, and pH, with initial values determined by preliminary experiments based on literature reviews (Table 1). The root length was used a dependent variable on the *A. cepa* test. Three assays on center point were added to the matrix in order to determine the standard error (Table 1). For each assay, three 5 mm diameter discs of the I.17 strain were used as inoculum in flasks containing 50 mL of culture media according to the test delimited by the matrix. The cultivation was conducted at 28 °C on 150 rpm for seven days.

**Table 1:** Fractional factorial design 2^5−1^ matrix and experimental data for treatment of tobacco extract from cigarrete wastes by *Fusarium* sp. strain I.17. Root length by *A. cepa* test to evaluate toxicity as an independent variable. The numbers in parentheses represent the levels studied for each factor.

Runs	Tobacco extract % (v/v)	Yeast extract % (v/v)	Dextrose % (v/v)	Copper sulfate (μmol.L^1^)	pH	Root length (mm)
1	15 (-1)	0,5 (-1)	0,5 (-1)	0 (-1)	8 (1)	0
2	25 (1)	0,5 (-1)	0,5 (-1)	0 (-1)	6 (-1)	0
3	15 (-1)	1,5 (1)	0,5 (-1)	0 (-1)	6 (-1)	0
4	25 (1)	1,5 (1)	0,5 (-1)	0 (-1)	8 (1)	0
5	15 (-1)	0,5 (-1)	1,5 (1)	0 (-1)	6 (-1)	0
6	25 (1)	0,5 (-1)	1,5 (1)	0 (-1)	8 (1)	0
7	15 (-1)	1,5 (1)	1,5 (1)	0 (-1)	8 (1)	0
8	25 (1)	1,5 (1)	1,5 (1)	0 (-1)	6 (-1)	0
9	15 (-1)	0,5 (-1)	0,5 (-1)	10 (1)	6 (-1)	4,47 ± 1,5
10	25 (1)	0,5 (-1)	0,5 (-1)	10 (1)	8 (1)	0
11	15 (-1)	1,5 (1)	0,5 (-1)	10 (1)	8 (1)	0
12	25 (1)	1,5 (1)	0,5 (-1)	10 (1)	6 (-1)	0
13	15 (-1)	0,5 (-1)	1,5 (1)	10 (1)	8 (1)	7,66 ± 1,6
14	25 (1)	0,5 (-1)	1,5 (1)	10 (1)	6 (-1)	13,69 ± 1,1
15	15 (-1)	1,5 (1)	1,5 (1)	10 (1)	6 (-1)	0
16	25 (1)	1,5 (1)	1,5 (1)	10 (1)	8 (1)	0
17	20 (0)	1 (0)	1 (0)	5 (0)	7,5 (0)	0
18	20 (0)	1 (0)	1 (0)	5 (0)	7,5 (0)	0
19	20 (0)	1 (0)	1 (0)	5 (0)	7,5 (0)	2,85 ± 1,2

To results analyze, the standardized effect was based on the following first-order polynomial model:

(1) y = β_0_ + ∑β_ixi_

Where y was the predicted response, β0 was the model intercept and βi was the linear coefficient and xi was the independent variable level.

Following the strategy based on the results of fractional factorial design the experiment was further expanded to a central composite design CCD (Table 2) with two factors: yeast extract and copper sulfate. A 2^2^ randomized factorial central composite design (CCD) with four star points α = (2^2^) ^¼^ and three replicates at the center points leading to a total of 11 experiments were employed to optimize the toxicity treatment of tobacco extract from cigarette wastes by strain I.17. The other three variables (dextrose, tobacco extract and pH) were fixed at the central levels of the fractional factorial experiment (Table 1). The levels to the independent factors, yeast extract, and copper sulfate, were established according to Table 2; the inoculum and cultivation were of the same fractional factorial design.

**Table 2:** Central composite design 2^2^ matrix and experimental data for treatment of tobacco extract from cigarette wastes by *Fusarium* sp. (I.17). Root length by *A. cepa* test to evaluate toxicity as an independent variable. The numbers in parentheses represent the levels studied for each factor.

Runs	Yeast extract % (v/v)	Copper sulfate (μmol.L^1^)	Root length (mm)
1	0,15 (-1)	6,45 (-1)	10,7 ± 1,9
2	0,85 (1)	6,45 (-1)	0
3	0,15 (-1)	15 (1)	0
4	0,85 (1)	15 (1)	34,4 ± 4,3
5	1,0 (1,41)	10 (0)	0
6	0,0 (-1,41)	10 (0)	0
7	0,5 (0)	15 (1,41)	11,6 ± 3,3
8	0,5 (0)	5,0 (-1,41)	8,6 ± 1,8
9	0,5 (0)	10 (0)	25,2 ± 5,7
10	0,5 (0)	10 (0)	54,3 ± 9,0
11	0,5 (0)	10 (0)	42,1 ± 1,8

These experiments were performed to obtain a second-order model to predict the percentage of root length on the functions of different variables. The quadratic model for predicting the optimal point was expressed as follow:

(2) y = β_0_ + ∑β_ixi_ + ∑β_iix_^2^i+ ∑β_ijxixj_

Where y was the predicted response, β_0_ was the model intercept, xi and xj were the independent factors levels and βi. βii. and βij were the linear quadratic and interaction coefficients, respectively.

The quality of fit of the model equation was expressed by the coefficient of determination R^2^, and its statistical significance was determined by F test (analysis of variance—ANOVA).

The results were analyzed by the software STATISTICA v.10. A significant level of 10% (p > 0.1) was considered for the variables screened, and it was 5% (p > 0.05) for the central composite design. To confirm the model equation adequacy, confirmatory experiments under the optimized condition were carried out (5% yeast extract and 10 μmol.L^−1^ copper sulfate). All the confirmatory experiments were conducted in triplicate, and the values predicted by the optimization model were set as controls.

### 2.3 Toxicity assay

The toxicity decrease of tobacco extract in the supernatant from cultivated fungi was evaluated using the *Allium cepa* (onion) test, according Arraes et al. (2012). Equal sized bulbs of *A. cepa* were purchased from the local market. Dried bulb onions and/or those with mold attack indication were discarded. The onions were submerged in the fungal culture supernatant (approximately 50 mL) for rooting. The experiment was performed at 24 ± 2 °C for three days. After 72 h, the root length was measured using a ruler, and the root tips were cut for later mitotic index determination. Filtered water and YDF media (without fungal culture) were used as positive and negative control, respectively. All the assays were carried out in triplicates.

### 2.4 Mitotic index (MI) determination

To analyze the efficiency of tobacco toxicity treatment, the mitotic index from the onions roots tips was calculated for each treatment according to Olorunfemi et al. (2012), with some modifications as follow: Root tips from each treatment were cut and fixed in ethanol: glacial acetic acid (3:1 v/v) and kept in 4 °C for 24 h before use. Then, the fixed onions tips were hydrolyzed in 1N HCl at room temperature for 10 min. After that, the hydrolyzed roots tips were squashed on a slide and stained with aceto-orcein for 10 min. A total of 1000 cells from four slides per sample were observed under 1000 x magnification using a Zeiss binocular light microscope Standard R.A. The MI was calculated according to the following equation:

$$Mitotic\;index\;\left(MI\right)=\;\frac{Number\;of\;dividing\;cells}{Total\;number\;of\;cells\;counted\;}\;\times 100$$

### 2.5 Laccase assay

Laccase activity was determined spectrophotometrically by monitoring the oxidation of 2,2-azino-bis-[3-ethyl benzothiazoline- 6-sulphonic acid] (ABTS), according Bonugli-Santos et al. (2016). The blank contains all of the constituents except the active enzyme. The activity was determined using the equation described in Baltierra-Trejo et al. (2015), and laccase was expressed as enzyme unit per liter, i.e. (μmol min^−1^) L^−1^.

### 2.6 Taxonomic characterisation

The isolate I.17 was cultured onto routine liquid culture media (Potato dextrose) and incubated at 28 °C until the earliest visible signs of growth were noted. A small amount (approximately 2 mm) of fungal mycelial mass was removed, and DNA was extracted according to Raeder and Broda (1985). Samples were used for polymerase chain reaction (PCR) and stored at -20 °C. The PCR reaction consisted of 5 μl (20 ng) of fungal DNA added to the mix with a final volume of 25 μl, composed of 2.5 μL Buffer 10x Taq polymerase, 0.75 μL MgCl2, 0.5 μL dNTPs, 1.25 μL each primer (10 mM) ITS1 (5′TCCGTAGGTGAACCTGCGG3′) and ITS4 (5′TCCTCCGCTTATTGATATGC3′),White et al. (1990), 0.2 μl of Taq DNA polymerase (Invitrogen of Brazil) and ultrapure water to complete the reaction volume. The amplification reactions were performed in Thermal Cycler Advance B960 under these thermal conditions: initial denaturation step (5 min at 94 °C), 30 cycles (30 s. at 94 °C, 1 min 30 s at 55 °C, 2 min at 72 °C) and final elongation step (5 min at 72 °C). At the end of the reaction, the product was stored at - 20 °C. Amplified fragments and control were separated by 1 % (w/v) agarose gel stained with gel red (Biotium) and visualised under UV light. The DNA was quantified in the Nano Drop 3300 spectrophotometer (Thermo Scientific). The PCR products were enzymatically cleaned before cycle sequencing by the addition of 3 μL of the ExoSAP-IT (USB Corporation, Cleveland, OH, USA) to 5 μL of amplified PCR product. The mixture was incubated at 37 °C for 30 min followed by 15 min at 80 °C. The PCR-amplified product was subjected to Sanger sequencing (ACTGene molecular analyses Company-Brazil).

The accuracy of the nucleotide sequence was achieved by performing two-directional DNA sequencing. The nucleotide sequences were compared to the National Centre for Biotechnology Information (NCBI) databases using the Blast search algorithm (Altschul et al., 1997). The sequences with the best combinations of explosions were recovered and integrated into the phylogenetic analysis. The sequences were aligned using Clustal-X and BioEdit 7.0, and analyzed using Mega-X software (Kumar et al., 2018), using Kimura's DNA substitution model (Kimura 1980). The phylogenetic tree was constructed using the neighbour-joining (NJ) (Felsenstein 1985; Saitou and Nei 1987), with bootstrap values calculated from 1,000 replicate runs.

## 3 Results and discussion

### 3.1 Screening of filamentous fungi able to grow and reduce toxicity of tobacco extract

To evaluate the potential of filamentous fungi in the toxicity treatment of tobacco extract from cigarette waste 38 fungi from CCMIBA were cultivated in the YDF culture media. Only fungus strain I.12 had the growth inhibited in tobacco extract, suggesting that the I.12 strain has not the ability to tolerate the tobacco toxicity. In contrast, all the other 37 strains showed satisfactory growth ranging from 1.74 g.L^−1^ to 5.57 g.L^−1^ (Supplementary material Table 1). The tobacco extract added in the culture media contains at least three contaminants in high concentrations that are worth mentioning due to their mutagenic capacity and also their potential to cause great environmental impacts (Trilha, 2009). These are nicotine, Polycyclic Aromatic Hydrocarbon - PAH (mainly Benzo[a]pyrene), and heavy metals, such as zinc, lead, and cadmium (Azevedo et al., 2013; Rosa, 2013; Trilha, 2009). Therefore, the tolerance to the media containing tobacco extract from cigarette wastes shows the potential of using fungi in bioremediation processes. According to Gurusamy and Natarajan (2013), the use of microorganisms for bioremediation is a promising method for recovering contaminated environments. The authors also describe the ability of microorganisms, such as fungi and bacteria, to use nicotine as a source of carbon and nitrogen for their growth in contaminated soils. The tolerance of fungi to tobacco extract was also studied by Pedroso (2003) for the strain *Pleurotus sp*., of which the author considered only the growth capacity in the presence of tobacco extract as a method for the study (Pedroso, 2003). Moreover, the efficiency of *Aspergillus terreus, Penicillium* sp*., Fusarium oxysporium* and *Trichoderma harzianum* to biodegrade organophosphorus pesticides is already known (Ramos, 2014; Hussaini et al., 2013), as well as the utilization of *Aspergillus* sp. in the biosorption of cadmium (Pallu, 2006), which are compounds that might also be present in the tobacco extract.

Although 37 fungal strains were capable of growing in a culture media containing tobacco extract, only the culture supernatant from the fungus *Fusarium* sp. strain I.17 allowed onion germination (Supplementary material Table 1). None of the other culture supernatants made the germination of the onions possible. The results suggest that even though the majority of fungal strains tested showed resistance/tolerance to the tobacco toxicity, these fungal strains are not capable of decreasing cigarette toxicity. Even supposing that the fungal strains might have been using nicotine as carbon and nitrogen sources (Gurusamy and Natarajan, 2013), other substances contained in the culture media, including biodegraded PAH molecules (Trilha, 2009) or heavy metals, may be interfering with the germination of onions, or still, the reduction of these contaminants was not sufficient enough to enable onions to root. In this sense, the performance showed by the *Fusarium* sp. strain I.17 was noteworthy in that it was able to reduce the toxicity of this variety of contaminants to a tolerable level that allowed the germination of onions. The *A. cepa* test is an ideal bioindicator for the first screening of toxicity assays due to its low cost, reliability, easily and quickly reproducible results. Furthermore, it is precisely the part of the plant that comes into contact with soil or water pollutants. Therefore, the growth pattern or even the inhibition of root growth are the first parameters to be considered (Leme and Marin-Morales, 2009). Another study has demonstrated the *A. cepa* efficiency as an organism to perform bioassays for detecting toxic effects induced by contaminants (Mazzeo et al., 2015).

### 3.2 Experimental design and optimization of *Fusarium* sp. strain I.17 to decrease cigarette toxicity

An experimental design was applied to optimize the performance shown by the *Fusarium* sp. (I.17) regarding its capacity for decreasing cigarette toxicity. We evaluated whether the fungus's growth in the culture medium with the different concentrations of the variables resulted in a supernatant with less toxicity. In the first step of these analyses, the influence of five independent factors in decreasing cigarette toxicity was investigated using Fractional Factorial Design 2^5−1^. The supernatants from these assays were evaluated in the toxicity assay *(A. cepa*), and results are shown in Table 1. The root length ranges were from 2.85 mm to 13.69 mm. The effects of the variables on the response (root length) and significant levels (p < 0.05) are shown in Table 3. Based on the statistical analysis, the variables that had the significant impact were yeast extract and copper sulfate. The yeast extract variable showed a negative effect on the response; in other words, the lowest concentrations (0,5 % v/v) used were the runs with higher root growth. Conversely, the copper sulfate variable was important in its high concentration (10 μmol.L^−1^), showing that higher levels could indicate improvements in the studied response.

**Table 3:** Effect of the variables on fractional factorial design 2^5−1^ in the root length by *A. cepa* test to evaluate toxicity in the treatment of tobacco extract from cigarrete wastes by *Fusarium* sp. strain I.17. The factors with significance (p-value < 0.05) are indicated by asterisk.

Factors	Effect	Standard error	p-value
Mean/Interc.	1,5092	0,3384	0,0210
(1)Tobacco extract	0,1955	0,7376	0,8081
(2)Yeast extract	-3,2280	0,7376	0,0221∗
(3)Dextrose	2,1105	0,7376	0,0645
(4)Copper sulfate	3,2280	0,7376	0,0221∗
(5)pH	-1,3130	0,7376	0,1731

It was possible to identify the variables that were important for the process, besides being possible to infer their trends, positively or negatively. In this sense, the significant variables were selected for the CCD, according to Table 2. The non-significant variables were maintained at the central levels, according to Table 1. According to the results shown in Table 4, yeast extract negatively influenced the toxicity decrease (assays 2, 3, 5 and 6, Table 2), inhibiting the onion roots. Thus, it shows the best results at the central level (0.5% v/v). To the copper sulfate variable, the best result was also at the central level (10 μmol.L^−1^). In statistical analyses, both variables tested were considered significant (p < 0.05) and are shown in Table 4. The multiple regressions from the results obtained (Table 4) led to the proposition of a mathematical model according to the significant variables, yeast extract and copper sulfate. The equation of the parametrized model is shown below:

$$y=41-19yeast\;extract^{2}-14copper\;sulphate^{2}$$

Where y is the predicted response (average root length in mm).

**Table 4:** Regression Coefficient obtained from CCD 2^2^ in the root length by *A. cepa* test to evaluate toxicity in the treatment of tobacco extract from cigarrete wastes by *Fusarium* sp. strain I.17. The factors with significance (p-value <0.05) are indicated by asterisk.

Factors	Regress	p-value
**Mean/Interc.**	40,5645	0,001510
Yeast extract (L)	2,9726	0,487216
Yeast extract (Q)	-18,7898	0,010624∗
Copper sulfate (L)	3,5047	0,417249
Copper sulfate (Q)	-13,6618	0,034293∗
1L by 2 L	11,3025	0,099559

The statistical significance (mathematical model) was checked by F test (ANOVA). As the F test value (7.31) for the regression was significant [higher than the F tabulated (4.46)], shown in Table 5.

**Table 5:** Analysis of variance (ANOVA) for root length by *A. cepa* test to evaluate toxicity in the treatment of tobacco extract from cigarrete wastes by *Fusarium* sp. strain I.17 obtained from CCD 2^2^.

Source of variation	Sum of squares	Degree of freedom	Mean squares	F-cal	F-tab	p-value
Regression	2388,04	2	1194,02	7,311	4,46	0,01563997
Residual	1306,507	8	163,32			
Total	3694,547	10	369,46			

**Figure 1:**
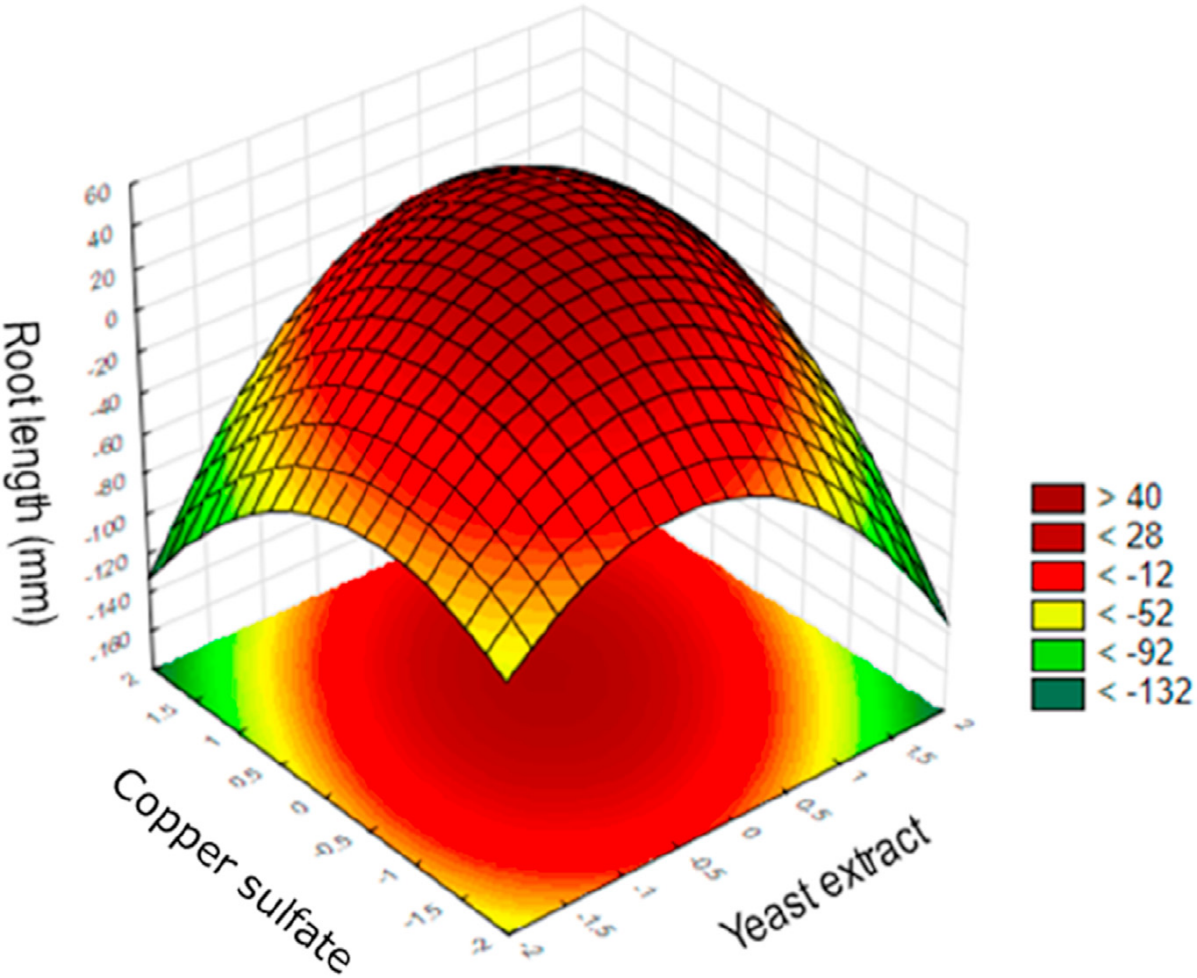
Response surface curve of the quadratic model to yeast extract and copper sulfate obtained from CCD 2^2^ in the root length by *A. cepa* test to evaluate toxicity in the treatment of tobacco extract from cigarette wastes by *Fusarium* sp. strain I.17. Legend represents the values (mm) of root length.

The percentage of variation was explained by the model 0.64 (R^2^ = 64%) and cannot be highly regarded, since only 64% of the mathematical model is explained. The lack of fit value is mainly due to the absence of root growth in some trials, and the variations found in the central points. According to Bonciu et al. (2018) the roots growth can be impacted by several factors that reflect in the variation of the root elongation. Moreover, the low regression coefficient obtained was an indication that the levels of yeast extract and copper sulfate concentrations used were not large enough to detect large variations in root length, possibly because the good results were at the central points. The results derived from the response surface (Figure 1) show that the study was close to the optimization point by using 0.5% of yeast extract and 10 μmol.L^−1^ of copper sulfate.

Fungi produce a myriad of extracellular enzymes of interest for bioremediation, such as lignin peroxidase and polyphenol oxidase (Shi et al., 2017; Rudakiya et al., 2019). Regarding PAHs degradation, two types of fungi metabolism are studied for bioremediation purposes, which are ligninolytic and non-ligninolytic fungi. The first one can mineralize PAHs due to their ability to produce ligninolytic enzymes (laccase, peroxidase – LiP, and manganese peroxidase – MnP). The enzymes laccase, LiP, and MnP are secreted extracellularly and oxidize the organic matters via non-specific radical-based reaction. The PAHs degradation occurs via hydrogenation of the aromatic ring, under anaerobic condition. Conversely, the mechanism of PAHs metabolism by non-ligninolytic fungi involves the oxidation of aromatic ring using Cytochrome P_450_ monooxygenase enzyme to yield arene oxide (Rudakiya et al., 2019). In this context, copper sulfate was added to induce some enzyme responses, especially as a cofactor. Since ligninolytic fungi have been reported in similar studies (Pedroso, 2003), laccase evaluation was performed, but there was no activity in our study.

Regarding nicotine degradation, most of what is known about microbial pathways were described to bacteria. The most known nicotine degradation pathways are pyridine and pyrrolidine pathways, in which reaction starts with hydroxylation (nicotine dehydrogenase) of the pyridine ring and oxidation (nicotine oxidase) of a carbon-nitrogen bond in the pyrrolidine ring, respectively (Gurusamy and Natarajan, 2013). However, the fungal metabolism of nicotine is less understood. Studying the metabolite routes involved in the process would require the development of advanced metabolomics and transcriptome methodologies, aiming to report the metabolites produced with the genetic pathways expressed by the fungus. Future studies can evaluate this process based on our results, which will be of vast scientific knowledge.

Another significant variable for the optimization of cigarette toxicity decrease was the presence of yeast extract in the culture media. Yeast extract provided a source of easy nitrogen assimilation for fungal growth. It was cited by Wang et al. (2009) as an important factor in nicotine degradation by possibly inducing the enzymatic route responsible for nicotine degradation. The results of the present study (Table 2 and Figure 1) indicate that 0.5% of yeast extract in the culture media is the maximum concentration to achieve the best result of reducing toxicity. According to Gurusamy and Natarajan (2013), nicotine degraded by fungi is used as a source of nitrogen. Therefore, increasing the availability of easily assimilated nitrogen (yeast extract) makes it advantageous for fungi to use this nitrogen source instead of using the enzymatic route to degrade nicotine. However, the results achieved suggest that a minimum source of easy nitrogen assimilation is needed, which agrees with the literature, as it was mentioned by Wang et al. (2009).

**Figure 2:**
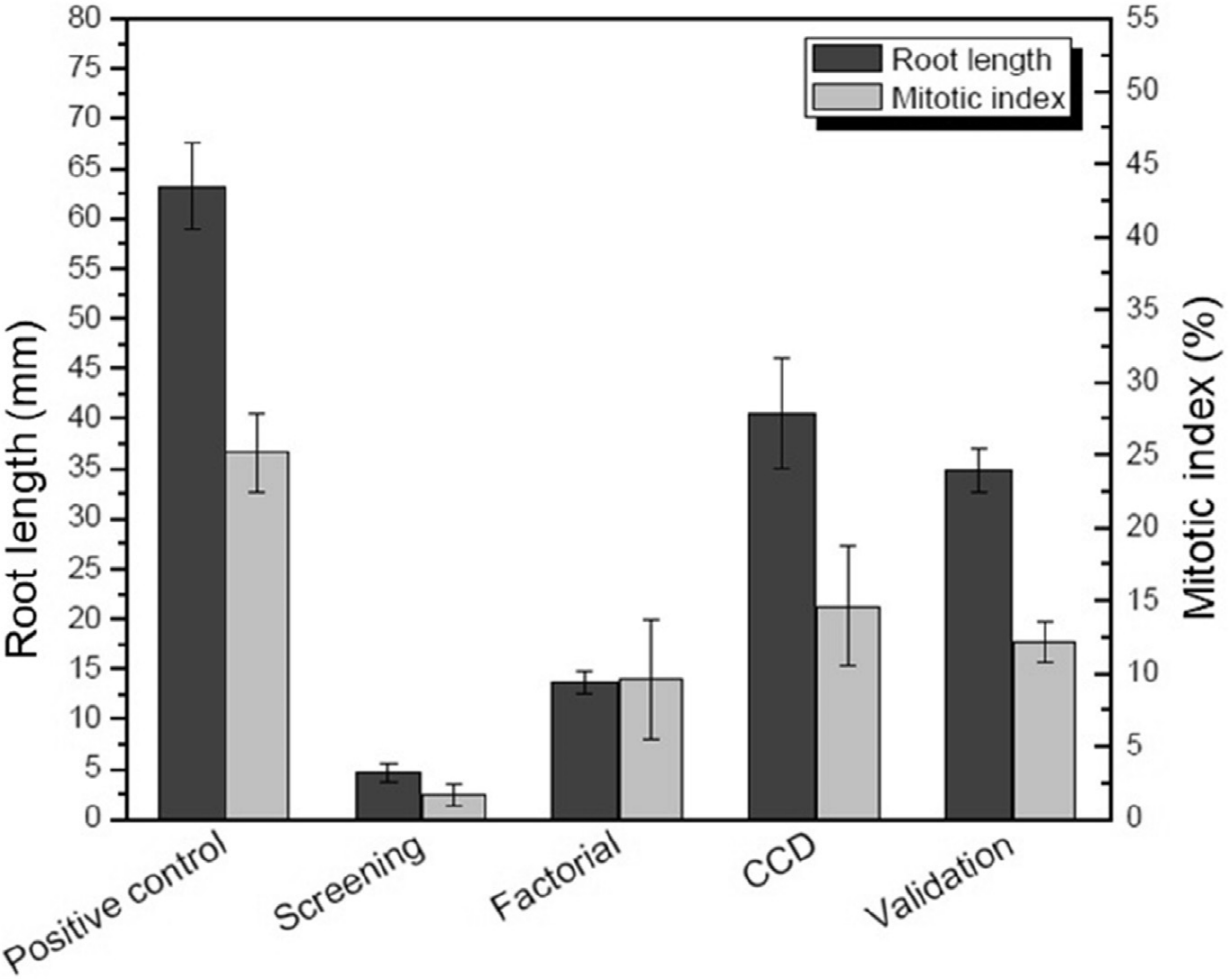
Treatments of tobacco extract from cigarette wastes by *Fusarium* sp. strain I.17, evolution by experimental design. Root length and Mitotic index by *A. cepa* toxicity test from screening, fractional factorial design 2^5−1^, CCD 2^2^, experimental design validation and positive control (root growth in filtered water in the validation assay). The experimental design validation represents 85% of the predicted value by the model equation from CCD 2^2^.

To finalize the experimental design an experiment under the optimized conditions was carried out to confirm the model equation (Figure 2). The result in triplicate reached 34.8 ± 2.2 mm of root length, representing 85% of the predicted value by the model equation (40.5 mm). Thus, the model was confirmed experimentally with a high percentage of predictability. Besides that, before the optimization process, the response (root length) achieved was 4.77 mm, representing 7.5% related to the control, and the strategy applied for optimization improved the response about seven times, reaching 34.8 mm, with 55% related to the control from the validation test (Figure 2).

### 3.3 Mitotic index

In addition to the root size, the mitotic index (MI) from the onions roots tips was calculated in all assay. The lower MI presented by all treatments than the positive control (Figure 2) is strong evidence of tobacco toxicity. The toxicity levels of a pollutant can be inferred by increasing or decreasing in the mitotic activity. MIs, notably lower than control, might indicate alterations in the growth and development of exposed organisms. Conversely, MIs higher than control can indicate a disordered cell proliferation, which is harmful to cells and could even drive in the formation of tumors (Leme and Martin-Morales, 2009). In this work, the mitotic activity, expressed as the mitotic index, was progressively improving by the evolution of the optimization methodology (Figure 2). Moreover, by comparison between the performances of *Fusarium* sp. strain I.17 before (screening – MI = 2,7 ± 0,8) and after (validation – MI = 12,2 ± 1,4) experimental design strategy had been applied shows an improvement to the cigarette toxicity treatment process. In this context, this work had not only identified a potential fungal strain to reduce cigarette waste toxicity but also had presented optimized culture media conditions to reach the best results.

The *A. cepa* MI is an acceptable and a standard measure of cytotoxicity environmental monitoring (Fiskesjo, 1985). According to Olorunfemi et al. (2012), MI is considered to trusty identify the presence of cytotoxic pollutants in the environment. *A. cepa* has been used worldwide since 1938 when this test system was introduced by Levan (1938). Since then, many technical modifications in the *A. cepa* test have been made to enable a more comprehensive assessment of environmental pollutants, but even nowadays, this technique has been considered to perform highly sensitive to identify and monitor pollutants in the environment. Fatima and Ahmad (2006) compared the genotoxicity of industrial wastewater from Aligarh and Ghaziabad cities by using the Ames plate incorporation test, the Ames fluctuation test and the *A. cepa* test. The authors concluded that all the test systems selected by them showed the same result, indicating that the *A. cepa* test can be reliably used. The authors also argued that in some comparisons between these three testing systems, the *A. cepa* had an advantage over the Ames test.

### 3.4 Phylogenetic analysis taxonomic characterization of strain I.17 and phylogenetic analyses

**Figure 3:**
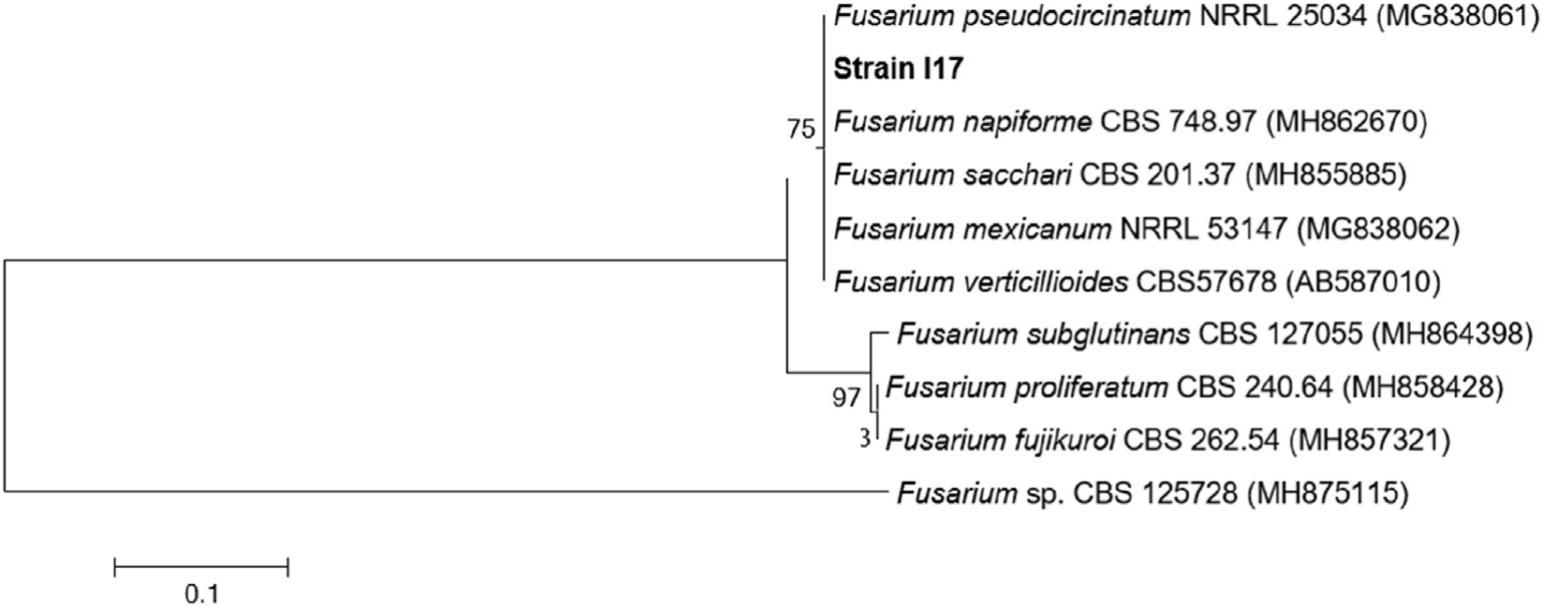
Phylogenetic analysis based on partial fungal ITS rRNA sequences from fungus strain I.17 and related species. Bootstrap values were made by 1,000 replicate runs (shown as %, greater than 70% are listed). GenBank accession numbers are listed after species names.

The fungus strains I.17 was identified by molecular methods. A 400-bp fragment containing ITS1, 5.8S rDNA from I.17 was sequenced and compared with sequences from NCBI database. The isolate showed a similarity of 99.5% with different species of *Fusarium* genus, family Nectriaceae, ordem Hypocreales, class Sordariomycetes and phylum Ascomycota (supplementary material Table 2). ITS sequences of I.17 were aligned with the consensus region and phylogenetic analysis maintained the same profile (Figure 3).

Morphological identification of *Fusarium* spp. is largely based on macro- and micro-morphological features, but successful identification may be difficult because of similar, inconspicuous or degraded diagnostic characters in culture. Though ITS has become the most sequenced region for identifying fungal taxonomy at species level and even within species, these regions do not work well for some species, and *Fusarium* spp. especially, due to the presence of two non-homologous copies within the region, causing divergence in results (Waalwijk et al., 1996).

*Fusarium* is found in plants and soils with a worldwide distribution and is an important animal, human, and plant pathogen. On the other hand, several studies are showing the capability of *Fusarium* strains to tolerate and transform pollutant molecules into non-contaminants intermediates. Chulalaksananukul et al. (2006) isolated one lineage of *Fusarium* sp. from *Pterocarpus macrocarpus Kurz* leaves and showed the ability of this lineage for benzo[a]pyrene biodegradation. Waszak et al. (2015) also showed the potential of *Fusarium* sp. for bioremediation works by applying a microbial consortium with *Pseudomonas aeruginosa*, *Candida albicans* and *Aspergillus flavus* to PAHs bioremediation, highlighting the possibility of successfully using *Fusarium* strains in microbial consortium for bioremediation purposes as well. Shi et al. (2017) demonstrated that *Fusarium* sp. can degrade PAH in contaminated soils. Their findings suggest that activities of lignin peroxidase and polyphenol oxidase are contributing to the degradation of PAH. However, the specific chemical mechanism still unknown requiring further studies. Cai et al. (2007) investigated a *Fusarium* strain for application in the remediation of a contaminated phenol environment, such as industrial wastewater. In literature, it is also mentioned that *Fusarium* sp. strains are able to tolerate and remove heavy metals from soils (Asra and Sashirekha, 2016). In a contaminated environment with toxic heavy metals, fungi are also frequently applied as an organism for bioremediation. *Fusarium solani* had shown a fast growth rate and higher capacity of copper and cadmium ion reduction (El-Sayed, 2014), and can be utilized as an eco-friendly alternative to the bioremediation of silver ions (El-Sayed and El-Sayed, 2020).

## 4 Conclusion

In the present study, 38 filamentous fungi strains were screened, regarding their capacity for reducing cigarette toxicity. Among them, only one strain had its growth inhibited. In contrast, the other ones showed dry weight ranging from 1.77 g.L^−1^ to 5.57 g.L^−1^, which might be a great source of enzyme screening for biotechnological application purposes. The fungus *Fusarium* sp. strain I.17 was capable of decreasing the toxicity sufficiently to enable the onion rooting. Finally, by applying an experimental design, it was possible to optimize its performance by about 14%, and the mathematical model has a high percentage of predictability. These findings may encourage new studies of biological activity by using a filamentous fungi isolate from cigarette waste to decrease cigarette toxicity and could provide a fungus strain (*Fusarium* sp. I.17) that can be applied in the bioremediation process, including in contaminated environments with cigarette waste and/or in the correct ecological destination of seized cigarettes. Further studies are necessary to confirm the toxic cigarette compounds reduced by the *Fusarium* sp. I.17 in the toxicity treatment, but the achievements of this study open up new avenues for cigarette waste.

## Declarations

### Author contribution statement

William Bartolomeu de Medeiros: Conceived and designed the experiments; Performed the experiments; Analyzed and interpreted the data; Contributed reagents, materials, analysis tools or data; Wrote the paper.

Jaqueline Bail: Performed the experiments; Analyzed and interpreted the data.

Michel Rodrigo Zambrano Passarini: Conceived and designed the experiments; Contributed reagents, materials, analysis tools or data.

Rafaella Costa Bonugli-Santos: Conceived and designed the experiments; Analyzed and interpreted the data; Contributed reagents, materials, analysis tools or data; Wrote the paper.

### Funding statement

This work was supported by Universidade Federal da Integração Latino-Americana (PAIP) (n ° 80/2019 / PRPPG).

#### Data availability statement

Data included in article/supp. material/referenced in article.

#### Competing interest statement

The authors declare no conflict of interest.

#### Additional information

No additional information is available for this paper.

## Appendix A Supplementary data

Supplementary materials_Medeiros et al_v26-08_final.docx Supplementary materials_Medeiros et al_v26-08_final.docx
