# Supplementary material for: Toxicity treatment of tobacco wastes using experimental design by filamentous fungi
Source: Heliyon. 2021 Feb 8;7(2):e06144. doi: 10.1016/j.heliyon.2021.e06144 (PMC7875829; doi:10.1016/j.heliyon.2021.e06144)
Supplement: Supplementary materials_Medeiros et al_v26-08_final.docx [file mmc1.docx]

**Supplementary material**

Supplementary table 1. Dry weight showed by the 38 fungi strains cultured in tobacco extract from cigarette wastes and the results from treatment of tobacco extract by these fungi in the toxicity evaluation using *A. cepa* test*.*

| **Fungal strains** | **Source** | **Dry weight (g/L)** | **Root length (cm)** |
| --- | --- | --- | --- |
| I.1 | cigarette | 5,35 ± 0,18 | __ |
| I.2 | cigarette | 4,39 ± 0,03 | __ |
| I.3 | cigarette | 4,84 ± 0,2 | __ |
| I.4 | cigarette | 4,17 ± 0,12 | __ |
| I.5 | cigarette | 3,43 ± 0,15 | __ |
| I.6 | cigarette | 1,74 ± 0,05 | __ |
| I.7 | cigarette | 3,7 ± 0,03 | __ |
| I.8 | cigarette | 3,72 ± 0,09 | __ |
| I.9 | cigarette | 2,41 ± 0,15 | __ |
| I.10 | cigarette | 2,61 ± 0,13 | __ |
| I.11 | cigarette | 4,11 ± 0,36 | __ |
| I.12 | cigarette | ___ | __ |
| I.13 | cigarette | 5,5 ± 0,17 | __ |
| I.14 | cigarette | 2,72 ± 0,08 | __ |
| I.15 | cigarette | 3,8 ± 0,15 | __ |
| I.16 | cigarette | 5,17 ± 0,61 | __ |
| I.17 | cigarette | 3,06 ± 0,21 | 4,7 ± 0,9 |
| I.18 | cigarette | 5,27 ± 0,13 | __ |
| I.19 | cigarette | 5,45 ± 0,13 | __ |
| I.20 | Iguassu National Park | 2,35 ± 0,12 | __ |
| I.21 | Iguassu National Park | 3,63 ± 0,2 | __ |
| I.22 | Iguassu National Park | 3,0 ± 0,1 | __ |
| I.23 | Iguassu National Park | 2,47 ± 0,08 | __ |
| I.24 | Iguassu National Park | 4,2 ± 0,18 | __ |
| I.25 | Iguassu National Park | 5,53 ± 0,07 | __ |
| I.26 | Iguassu National Park | 4,53 ± 0,09 | __ |
| I.27 | Iguassu National Park | 3,31 ± 0,37 | __ |
| I.28 | Iguassu National Park | 2,04 ± 0,21 | __ |
| I.29 | Iguassu National Park | 5,77 ± 0,15 | __ |
| I.30 | Iguassu National Park | 3,36 ± 0,06 | __ |
| I.31 | Iguassu National Park | 2,71 ± 0,18 | __ |
| I.32 | Iguassu National Park | 2,23 ± 0,17 | __ |
| I.33 | Iguassu National Park | 5,68 ± 0,11 | __ |
| I.34 | Iguassu National Park | 1,83 ± 0,07 | __ |
| I.35 | Iguassu National Park | 2,25 ± 0,07 | __ |
| I.36 | Iguassu National Park | 2,14 ± 0,1 | __ |
| I.37 | Iguassu National Park | 1,98 ± 0,03 | __ |
| I.38 | Iguassu National Park | 4,54 ± 0,11 | __ |
| Positive control |  | __ | 67,1 ± 5,2 |
| Negative control |  | __ | __ |

**Supplementary table 2.** Result from Blast search in the National Center for Biotechnology Information (NCBI) databases to strain I.17. Ten different taxa: % identity, accession code in the NCBI, and E value.

| **Genbank _NCBI** | | | |
| --- | --- | --- | --- |
| **% Identity** | **Accession** | **Taxa** | **E value** |
| 99.5 | MG838062 | *Fusarium mexicanum* NRRL53147 | 0.0 |
| 99.5 | MG838061 | *Fusarium pseudocircinatum* NRRL25034 | 0.0 |
| 99.5 | MH862670 | *Fusarium napiforme* CBS74897 | 0.0 |
| 99.5 | MH911404 | *Fusarium fujikuroi* MF22418 | 0.0 |
| 99.5 | MF411134 | *Fusarium verticillioides* ATS105 | 0.0 |
| 99.5 | MK226291 | *Fusarium proliferatum* strain TF1 | 0.0 |
| 99.5 | MK397501 | *Fusarium* sp. B7-1 | 0.0 |
| 99.5 | MH483991 | *Fusarium sacchari* BN2 | 0.0 |
| 99.5 | MG274315 | *Fusarium subglutinans* isolate 07038 | 0.0 |
| 99.5 | JX491141 | *Gibberella moniliformis* isolate GM-MSN17 | 0.0 |
